# Reporting of Hospital-Free Days As an Outcome Measure in Critical Care Trials: A Systematic Review

**DOI:** 10.1097/CCM.0000000000006858

**Published:** 2025-09-24

**Authors:** Ralph Shackleton, Sarah Vollam, Stephen Gerry, Akshay Shah, David M. Griffith

**Affiliations:** 1 Centre for Population Health Sciences, The University of Edinburgh, Edinburgh, United Kingdom; 2 Nuffield Department of Clinical Neurosciences, University of Oxford, Oxford, United Kingdom; 3 NIHR Oxford Biomedical Research Centre, Oxford, United Kingdom; 4 Centre for Statistics in Medicine, University of Oxford, Oxford, United Kingdom; 5 Hammersmith Hospital, Imperial College Healthcare NHS Trust, London, United Kingdom

**Keywords:** critical illness, follow-up, outcomes, systematic review

## Abstract

**Objectives:**

To synthesize the existing use, definitions, and variation in the application of hospital-free days (HFDs) as an outcome measure in randomized controlled trials (RCTs) of critically ill adults.

**Data Sources:**

Trial registries (ISRCTN, ClinicalTrials.gov) and electronic databases (CENTRAL, MEDLINE, Embase, and CINAHL PLUS).

**Study Selection:**

We included trial registrations, protocols, or articles reporting RCTs that included patients admitted to an adult ICU and had any variation of HFD as a primary or secondary outcome.

**Data Extraction:**

Data collected included definition of HFD, statistical analysis, minimal clinically important difference, method of data collection, and loss to follow-up. Risk of bias in the included studies was assessed using the relevant domains of the Cochrane Risk of Bias 2 tool (blinding of outcome assessment, incomplete outcome data, and selective reporting). Data were synthesized quantitatively using frequencies and percentages.

**Data Synthesis:**

We identified 110 eligible studies. We found considerable variability in how HFD was defined and reported. Incomplete reporting was common, with 69 studies (62.7%) not reporting all three individual components of HFD. Length of stay was omitted most frequently. Risk of bias related to outcome assessment and measurement was considered low. Fifty-two studies (47.3%) collected HFD data from routine healthcare records. The most common follow-up time points were 28 and 90 days. Over half of all studies (56 [50.9%]) did not report the number of HFD counted if a patient died during follow-up.

**Conclusions:**

This systematic review highlights the heterogeneity in the definition, reporting, and analysis of HFD. We propose guidance for the use of HFD and highlight areas for future research to allow standardization in the use and reporting of HFD in critical care research.

**KEY POINTS**

**Question**: How is hospital-free days (HFDs) defined, reported, and analyzed as an outcome measure in critical care research?

**Findings**: We included 110 studies. Despite increasing use as an outcome measure, we demonstrate substantial variation in the definition, reporting, and analysis of HFD.

**Meaning**: HFD is a feasible, objective, patient-centered outcome but further work is needed to standardize and report HFD in critical care research.

Hospital-free days (HFDs) is a count of all days spent outside of hospital after a specific hospitalized event. It is increasingly being used as an outcome measure in critical care and perioperative research (1, 2). Unlike mortality, HFD demonstrates resource use (hospital length of stay, readmissions) and discharge destination (2–4). It is associated with health-related quality of life (HRQoL) measures such as EuroQol-5D (EQ-5D) and 36-item Short-Form Health Survey Physical Component Summary at 90 and 180 days (4, 5). Additionally, HFD allows data from nonsurvivors to be incorporated (5, 6).

There are challenges in using HFD in critical care research. First, excessive variation in defining, calculating, and reporting HFD affects external validity (7–10). This largely arises from discrepancies in the inclusion of readmission, definition of a hospital day, handling of death, and duration of follow-up (2, 3, 7, 8, 11, 12).

Third, consensus on the minimal clinically important difference (MCID) and noninferiority margin (NIM) for HFD has not been reached (3, 13). Finally, HFD often has a bimodal distribution and therefore requires more complex statistical analyses that are not consistently applied (3).

Our systematic review aimed to understand how HFD has been used as an outcome measure in randomized controlled trials (RCTs) in critically ill patients. Specific research questions included:

1) How commonly is HFD used as an outcome measure in RCTs in critically ill patients?
2) How is HFD defined and how consistent are these definitions across RCTs?
3) How has data on HFD been collected and analyzed, including the estimation and reporting of MCID, NIM, and approaches to bimodal distribution of data?
4) Are there any recommendations to guide a standardized and robust framework for using HFD in critical care RCTs?

## MATERIALS AND METHODS

This review was designed, conducted, and reported according to the Preferred Reporting Items for Systematic reviews and Meta-Analyses (PRISMA) 2020 guidelines (**Supplementary Table S1**, https://links.lww.com/CCM/H794). Study methods followed a prospectively registered study protocol (CRD42023402958). Covidence software (Veritas Health Innovation Ltd, Melbourne, VIC, Australia) was used to import studies, identify duplicates, collect, and export data (14).

Eligible studies were RCTs enrolling patients admitted to an adult ICU that included any variation of HFD as a primary or secondary outcome. Eligible studies included pre-prints or published protocols of ongoing trials, published conference abstracts and articles, and unpublished trial registry entries. We included ongoing and unpublished trials to analyze the methodological aspects of HFD rather than final outcomes, which was beyond the scope of this review. We only included studies for which an English language version was available.

Studies were last searched for on April 1, 2024, on Embase, MEDLINE, CINAHL Plus, and CENTRAL. Additionally, we searched for planned, ongoing, and unpublished trials on ClinicalTrials.gov and ISRCTN. Reference lists from included studies were examined for other eligible studies. A subject specialist librarian was consulted on creating the search strategy (**Supplementary Material 1**, https://links.lww.com/CCM/H794).

Title, abstract, and full-text screening were performed independently by two reviewers. Disagreements were resolved through discussion. Multiple records corresponding to a single RCT were merged.

Data were extracted by one reviewer onto a pre-piloted data collection form using Covidence (**Supplementary Material 2**, https://links.lww.com/CCM/H794). If required, authors of RCTs were contacted to obtain further information using a piloted template.

Risk of bias in the included studies was assessed independently by two reviewers in accordance with guidance in the Cochrane Handbook and Cochrane Risk of Bias 2 tool (15). We assessed only the domains relevant to the aims of this review (blinding of outcome assessment, incomplete outcome data, and selective reporting).

No meta-analysis or measure of effect was performed.

## RESULTS

### Study Characteristics

**Figure 1.**
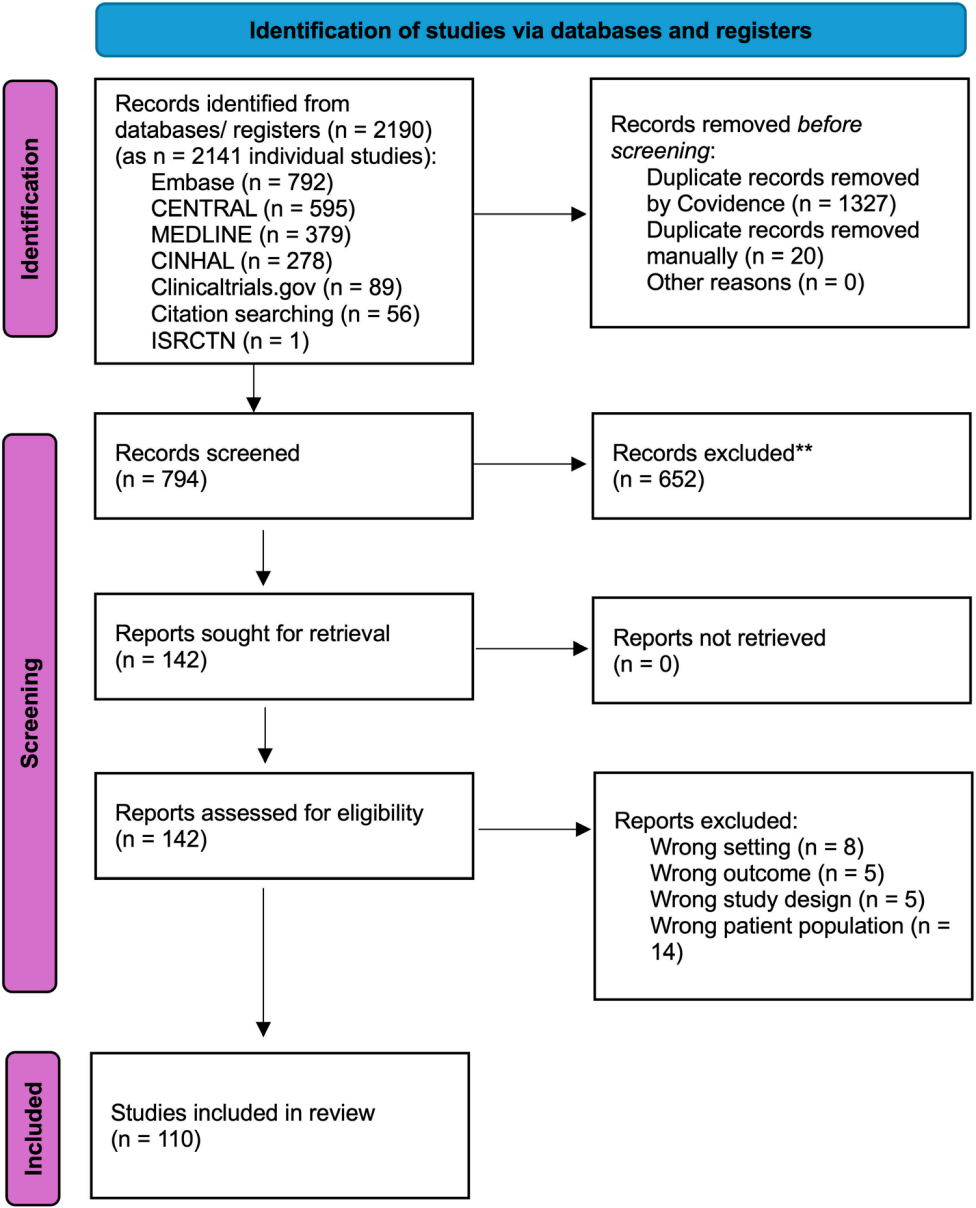
Preferred Reporting Items for Systematic reviews and Meta-Analyses (PRISMA) 2020 flow diagram. Adapted from Page MJ, McKenzie JE, Bossuyt PM, et al: The PRISMA 2020 statement: An updated guideline for reporting systematic reviews. *BMJ* 2021; 372:n71. Adaptations are themselves works protected by copyright. So in order to publish this adaptation, authorization must be obtained both from the owner of the copyright in the original work and from the owner of copyright in the translation or adaptation.

We identified 2190 references. After removal of duplicates, 814 references remained for title and abstract screening. One hundred forty-two articles were selected for full-text review and 110 studies were included (**Fig. 1**; and **Supplementary Table S2**, https://links.lww.com/CCM/H794).

All included studies were conducted between 2000 and 2024, with the majority since 2016 (83, 75.4%). Around two-thirds of all studies (70, 63.6%) evaluated a therapeutic intervention. Thirty studies (27.3%) evaluated a change in patient care, for example, a new ventilation or fluid strategy and 10 (9.1%) evaluated a medical device.

The planned or actual sample size of included studies ranged from 23 to 13,903. Twenty-four studies (21.8%) planned to recruit or recruited fewer than 100 patients, 37 (33.6%) between 101 and 500 patients, and 49 (44.6%) greater than 500 patients.

The included studies were generally judged to be at low risk of bias in our domains of interest but many of the included studies had not yet completed or reported their findings (**Supplementary Table S3**, https://links.lww.com/CCM/H794).

### Use and Terminology of HFD

We included 40 completed studies (36.4%) with published results (**Supplementary Table S4**, https://links.lww.com/CCM/H794), which included a total of 38,652 patients. There were also nine (8.1%) secondary analyses, 19 (17.3%) published protocols, and 42 (38.2%) trial registries. In total, 12 studies (10.9%) use HFD as a primary outcome (**Table 1**) and 98 studies (89.1%) as a secondary outcome. HFD was the most commonly used term followed by days alive out of hospital (**Supplementary Table S5**, https://links.lww.com/CCM/H794).

**TABLE 1.** Table of Included Studies With Hospital-Free Days As a Primary Outcome and Supporting Minimum Clinically Important Difference

Trial Name	MCID	Reference for MCID	Method of MCID
Early Active Mobilization during Mechanical Ventilation in the ICU (TEAM)	7 d	Pilot RCT (~half the point estimate in the pilot RCT)	Distribution-based
AID-ICU	8% (4.7 d)	AID-ICU cohort study (8% of baseline 59 HFD)	Distribution-based
Haloperidol vs. Placebo for the Treatment of Delirium in ICU patients: A Pre-Planned, Secondary Bayesian Analysis of the AID-ICU Trial	1 d	Not specified	Not specified
Goal-Directed Fluid Removal With Furosemide Versus Placebo in Intensive Care Patients With Fluid Overload: A Randomised, Blinded Trial (GODIF Trial-First Version)	8%	Not specified	Not specified
Furosemide Versus Placebo for Fluid Overload in Intensive Care Patients—The Randomised GODIF Trial Second Version: Statistical Analysis Plan	8% (4.7 d)	AID-ICU cohort study (8% of baseline 59 HFD in cohort study)	Distribution-based
Study Protocol for TARGET Protein: The Effect of Augmented Administration of Enteral Protein to Critically Ill Adults on Clinical Outcomes: A Cluster Randomised, Cross-Sectional, Double Cross-Over, Clinical Trial	1 d	Target calories RCT	Distribution-based
Iron and Erythropoietin to Heal and Recover After Intensive Care (ITHRIVE): A Pilot Randomised Clinical Trial	3 d	Secondary outcome in pilot RCT	Anchor-based
Plasma Exchange (PLEX) and Convalescent Plasma (CCP) in COVID-19 Patients with Multiorgan Failure (COVID-PLEX) (NCT04634422)	7.31 d	Unreferenced cohort study	Distribution-based
Early and Sustained Lactobacillus plantarum Probiotic Therapy in Critical Illness: The Randomised, Placebo-Controlled, Restoration of Gut Microflora in Critical Illness Trial (ROCIT)	4 d	Consumer and Community Health Research Network, University of Western Australia, WA	Consensus-based
Fibrinogen Early In Severe Trauma StudY II (FEISTY II) (NCT05449834)	Not specified	Not specified	Not specified
Balanced Multi-Electrolyte Solution Versus Saline Trial for Diabetic KetoAcidosis (BEST-DKA) (NCT05752279)	Not specified	Not specified	Not specified
Randomised, Controlled, Feasibility Trial Comparing Vasopressor Infusion Administered via Peripheral Cannula Versus Central Venous Catheter for Critically Ill Adults: A Study Protocol (VIPCA)	Not specified	Not specified	Not specified

AID-ICU = Haloperidol for the Treatment of Delirium in ICU Patients, HFDs = hospital-free days, MCID = minimum clinically important difference, RCT = randomized controlled trial.

### Reporting, Definitions, and Handling of Individual Components

HFD is a composite outcome of mortality, hospital length of stay, and readmissions. Six studies, all of which were trial registration entries, did not plan to report either mortality or hospital length of stay alongside HFD. Sixty-nine studies (62.7%) did not report hospital length of stay. One published protocol did not report mortality.

#### Mortality

Three studies (2.7%) assigned a value of –1 to death within the follow-up period, 32 (29.1%) assigned a value of 0, and one (0.9%) assigned a value of +1 (the author did not respond to a request to clarify this). Sixteen studies (14.5%) assigned the actual number of days a patient was alive and out of hospital before they died within the follow-up period, of which five (4.5%) censored follow-up at hospital discharge. One study (0.9%) assigned mortality the longest observed hospital length of stay value (16). Fifty-seven studies (51.8%) did not specify how they handled death, including four studies which plan to use HFD as a primary outcome.

#### Hospital Days

Thirty-four studies (30.9%) considered time spent in a nursing home, hospice, or rehabilitation facility as an HFD. Thirteen studies (11.8%) considered these days to be equivalent to time spent in hospital. Sixty-three studies (57.3%) did not specify this.

#### Readmission

Eighteen studies (16.4%) did not consider readmissions to hospital as they censored data collection at index hospital discharge. Thirty-four studies (30.9%) specified that readmissions were included in the calculation of HFD. Fifty-eight studies (52.7%) did not specify.

#### Follow-Up Time

Follow-up duration for HFD varied (**Table 2**). Ninety days was the most common duration followed by 28 days.

**TABLE 2.** Table of Follow-Up Durations for Hospital-Free Days

Duration	Frequency (%)
28 d	31 (28.2)
29 d	2 (1.8)
30 d	8 (7.3)
60 d	13 (11.8)
90 d	43 (39.1)
91 d	1 (0.9)
180 d	5 (4.5)
1 yr	2 (1.8)
2 yr	1 (0.9)
28 and 60 d	1 (0.9)
30 and 90 d	1 (0.9)
28, 90, and 180 d	1 (0.9)
Not specified	1 (0.9)

### Data Collection and Loss to Follow-Up

In total, 52 studies (47.3%) collected data on HFD from routinely collected healthcare records, 36 (32.7%) used telephone follow-up, 24 (21.8%) used national registries, five (4.5%) used in-person visits, and four (3.6%) contacted primary care physicians. Some 42 studies (38.2%) did not specify how data pertaining to HFD were collected. Loss to follow-up rates ranged from 0% to 28.4% (median, 1.1%).

### Statistical Analysis and Considerations

#### Minimal Clinically Important Difference and Noninferiority Margin

The MCID is defined as the smallest difference that is considered meaningful to patients when assessing the clinical utility of therapies intended to improve outcomes (17). The MCID was reported in nine of 12 studies with HFD as the primary outcome (Table 1; and **Supplementary Table S6**, https://links.lww.com/CCM/H794) and ranged from 1.0 to 7.3 days. NIM was not reported in any of the included studies.

#### Statistical Tests

Of the 62 studies that reported their statistical analysis, 25 studies used nonparametric tests, for example, Mann-Whitney *U*, van Elteren, or Kruskal-Wallis tests. Twenty-eight studies used models such as generalized linear regression or quantile regression models. Five studies used parametric tests, for example, two-sample *t* test, analysis of variance, analysis of covariance, Kryger Jensen, and Lange test. Four studies used Bayesian analysis (Supplementary Table S4, https://links.lww.com/CCM/H794).

## DISCUSSION

The key findings of this systematic review were: 1) there was substantial variation in how HFD was defined; 2) reporting of HFD and its composites was inconsistent; and 3) methods of statistical analysis used lacked description and justification. Additionally, we found a lack of stakeholder engagement (e.g., former patients, clinicians, policy makers) informing sample size calculations based on a clinically important difference.

On the basis of the findings of our review, we make a number of recommendations for the use of HFD in RCTs in critically ill patients (**Table 3**; and **Supplementary Table S7**, https://links.lww.com/CCM/H794). These recommendations are suggestions to be carefully considered when designing a future trial with an HFD outcome. There is unlikely to be a “one-size-fits-all” approach given the heterogeneity of the populations, healthcare systems, and interventions being studied.

**TABLE 3.** Abbreviated Recommendations for the Use of Hospital-Free Days in Critical Care Research (to Be Used in Conjunction With Supplementary Table S7 [https://links.lww.com/CCM/H794], Which Includes Justification)

Area	Recommendation
Definition and reporting	The HFD outcome should be clearly and prospectively defined in the trial registration or protocol of a study.
	Mortality and hospital length of stay in the same follow-up period should be reported.
Mortality	How mortality is used in the calculation of HFD should be prospectively specified.
	More patient and public involvement work is needed to consider different values assigned to mortality. In the meantime, we suggest that a value of 0 HFD be assigned.
Definition of a hospital-free day	What was counted as a “hospital-day” should be clearly specified.
	We suggest that time spent in an long-term acute care hospital, emergency department visits, and nursing homes be counted as hospital days.
	Authors should consider using institution-free days, which typically include outpatient visits and other healthcare encounters in the calculation.
Quality-weighted HFD	More work is needed to validate quality-weighted HFD.
Readmissions	We strongly recommend that follow-up should not be censored on discharge from the index hospitalization. An outcome that is censored on discharge from the index hospital admission should not be identified as HFD.
	We recommend that readmissions to an acute care hospital should be included in the calculation of HFD.
Follow-up duration	Follow-up duration should be sufficient to allow discharge from the index hospitalization for the majority of patients and to capture relevant readmissions.
MCID	The MCID should be prospectively specified and justified. More work is needed to consider the MCID at different follow-up durations.
Statistical analysis	We suggest that the analysis method used reflects the distribution of the HFD data and is described and justified in sufficient detail. More work in this area is required to establish the optimal methods of analysis.

HFDs = hospital-free days, MCID = minimal clinically important difference.

### Definition and Reporting

Our findings are broadly consistent with other published work. Ribeiro et al (18) recently reported on variation in the terms used, definitions applied, and reporting standards associated with the use of days at home after surgery as a perioperative outcome. One scoping review of nonmortality outcomes in RCTs of critically ill patients found that HFD was used in 18 trials, with notable inconsistency in the value assigned to deceased patients (9).

A different review of RCTs evaluating ventilator-free days identified similar variation in definitions. It also highlighted other issues, such as a lack of external validity by preventing direct comparisons between studies, problematic data synthesis in meta-analyses, and a greater risk of data bias if the outcome was not defined in the protocol. This review, and another, highlighted a need to establish standardized definitions for ventilator-free days and other free-days outcomes (7, 19).

**Figure 2.**
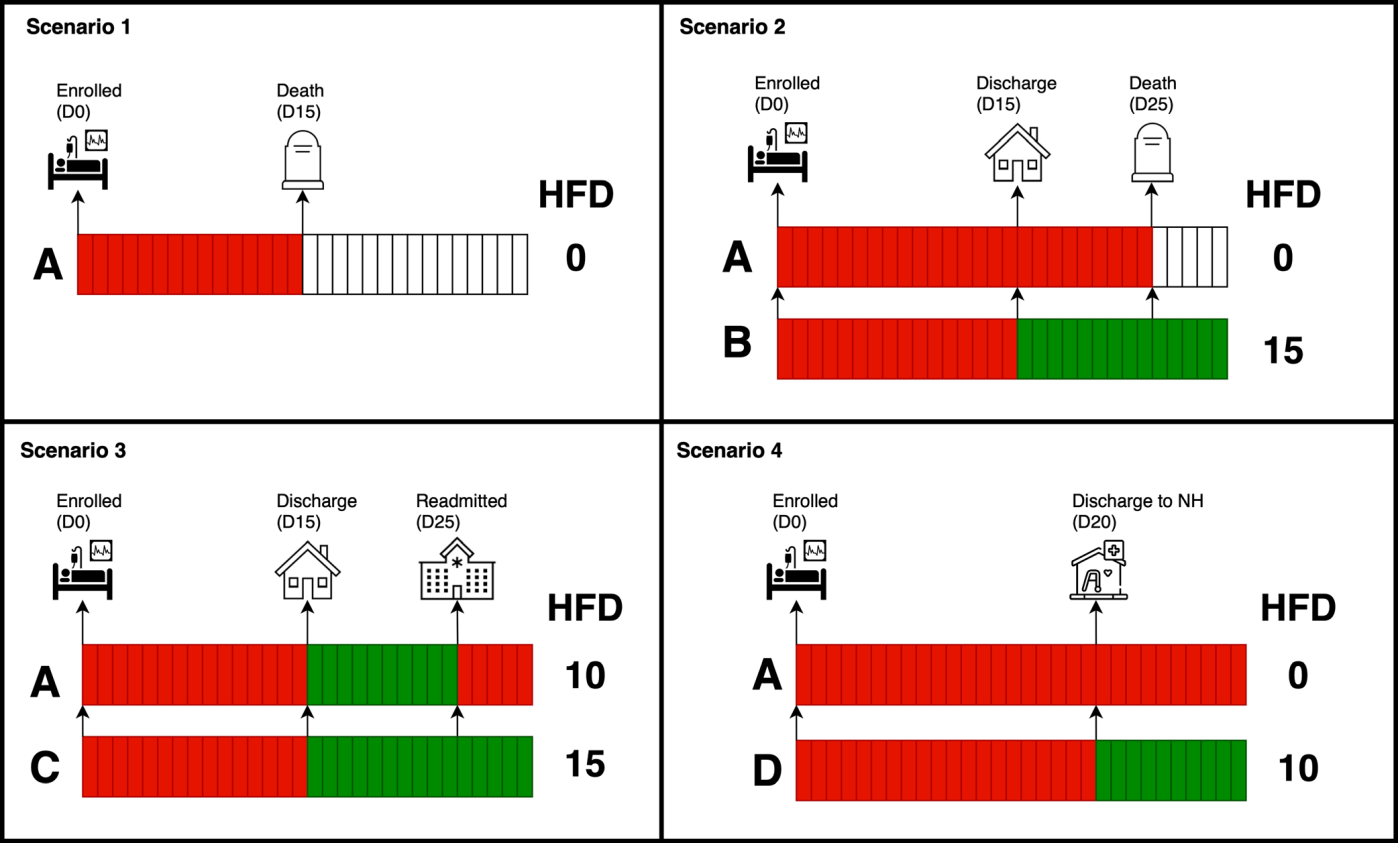
Variation in hospital-free days (HFDs; 30 d) calculation according to definition. Panels illustrate diﬀerent clinical journeys in a 30-d follow-up period. *Letters A–D* represent diﬀerent HFD definitions used to calculate HFD, the results displayed on the right. **Definition A**—Death at any time during follow-up is assigned 0 HFD. Follow-up is not censored at discharge from the index hospitalization. Readmissions to hospital are accounted for and not counted as HFDs. Days spend in a nursing home (NH; where not previously a resident), long-term acute care hospital (LTACH), or rehabilitation center are accounted and are not counted as HFDs. **Definition B**—Follow-up is censored at hospital discharge so information relating to events (e.g., readmission, death) are not captured. **Definition C**—Readmissions to hospital are not accounted for and counted as HFD. **Definition D**—Days in a NH (where not previously a resident), LTACH, or rehabilitation center is counted as HFDs. One *small box* represents 1 d in the 30-d follow-up period. A *red box* indicates a day which is not counted as an HFD in that definition, and a *green box* indicates a day which is counted as an HFD.

The differences in the definition, reporting, and analysis of HFD between two recent trials (Haloperidol for the Treatment of Delirium in ICU Patients [AID-ICU] and Early Active Mobilization During Mechanical Ventilation in the ICU [TEAM]) is demonstrated in **Table 4** (20, 21). **Figure 2** highlights how the definition can impact on calculating HFD.

**TABLE 4.** Table Comparing the Use of an Hospital-Free Days Outcome in Two Large, Multicenter Critical Care Trials

Trial Name	Haloperidol for the Treatment of Delirium in ICU Patients (AID-ICU)	Early Active Mobilization During Mechanical Ventilation in the ICU (TEAM)
Population	Adult patients in ICU with delirium	Adult patients in ICU undergoing mechanical ventilation
Intervention	Haloperidol	Early active mobilization
Comparison	Placebo	Usual care
Number of included participants	1000	750
Primary outcome	Days alive and out of the hospital	Days alive and out of the hospital
Follow-up duration	90 d	180 d
Components reported	Hospital length of stay, mortality	Mortality only
Mortality	Actual number of HFD used	Assigned 0 HFD
Readmissions	Counted as hospital days	Counted as hospital days
Definition of a hospital days	Days spent in an acute care hospital only	Days during the index hospitalization, hospital readmission, inpatient rehabilitation, or in a nursing home
Definition of a HFDs	Included time spent in a nursing home, rehabilitation facility, long stay hospital, or inpatient hospice	Home or in an accommodation that was not a healthcare facility
MCID	8%	7 d
Reference for MCID	AID-ICU cohort study	Half the point estimate found in the pilot randomized controlled trial
Statistical analysis	Linear regression model	Linear quantile regression analysis

AID-ICU = Haloperidol for the Treatment of Delirium in ICU Patients, HFDs = hospital-free days, MCID = minimal clinically important difference, TEAM = Early Active Mobilization During Mechanical Ventilation in the ICU.

### Mortality

An important consideration in the use of HFD is the handling of mortality. The majority of the studies in our review assigned a value of 0 HFD to mortality within the follow-up period. Several studies used a value of –1 HFD and some used the actual number of HFD for patients who died after discharge from the index hospitalization but still in the follow-up period.

In their recommendation of HFD at 90 days as an outcome in phase II trials in intensive care, the Australian and New Zealand Intensive Care Society (ANZICS) Clinical Trials Group consensus panel considered both assigning nonsurvivors the longest hospital length of stay or a value of zero. They felt the latter should be used to keep the outcome in line with definitions of ventilator-free days (22).

One retrospective cohort study compared three different definitions of institution-free days (IFDs), a variation of HFD discussed below—one where follow-up was censored at hospital discharge, one where mortality was assigned a value of 0, and one where mortality was not penalized. These were broadly similar to definitions we found in studies included in this review. It concluded that IFDs varied significantly with each of the definitions and that patient input was required to decide the most patient-centered variation (10).

The value used has statistical implications and can influence how patient-centered the outcome is. Assigning a value of 0 days for patients that die during follow-up assumes that death during follow-up is of equally poor value as spending the entire duration of follow-up in hospital (also given a value of 0 HFD). For this reason, death is sometimes assigned a value less than 0 (e.g., –1). This approach does not convey the potential value that patients often place on days at home before death.

In a survey of patients who received organ support for over 24 hours in six ICUs in Australia, survivors ranked severe disability requiring care and inability to return home (needing a nursing home or assisted living) as their top two of eight health concerns. Interestingly, concerns about dying received the lowest scores, although the study recognized that this may be attributable to survivor bias (12). Another small survey of adults with serious illnesses identified various physical, cognitive, and social impairments as states worth than death (23).

### Definition of Hospital Days

There is a distinction between time spent out with the acute hospital, which could include time at long-term acute care hospitals (LTACHs), or rehabilitation hospitals, nursing homes, and time spent at home, which is the patient’s residence before admission to the hospital (2). Most definitions of HFD counted a day in an LTACH, nursing home, or inpatient hospice as a HFD. It is clear from the survey discussed above that patients place considerable value on returning home (12). It is also important to consider that patients may find time spent in rehabilitation facilities as useful time.

IFD is a similar concept to HFD but typically includes outpatient hospital visits and other healthcare encounters as an institution day (10). Auriemma et al (23) gathered stakeholder perspectives on the definitions of HFD and IFD. Respondents were asked to categorize a day spent in 12 healthcare settings. A hospital day was always categorized as an institution day but not vice versa. Over half categorized a day spent in an LTACH or emergency department (ED) as a hospital day. Around three-quarters categorized inpatient rehabilitation, long-term nursing home, or skilled nursing facility as an institution day, and just over half felt that an inpatient hospice was an institution day. An overnight stay was identified as a key indicator of a hospital or institution day.

Auriemma et al (24) proposed that the working definition of a hospital day should include any part of a day spent in a short-term acute care hospital or LTACH or ED and that an institution day should additionally include any part of a day spent in inpatient rehabilitation or in a nursing home, skilled nursing facility, or inpatient hospice. The most important consideration in this area is consistency in terminology and justification for the definition used.

### Quality-Weighted HFD

It is also important to consider that time spent at home can vary in quality depending on a patient’s comfort, function and underlying comorbidities. Quality-weighted HFD (QW-HFD) is a further composite of total HFD in a follow-up period adjusted for a HRQoL measure, a concept similar to quality-adjusted life years. Auriemma et al (25) recently undertook a modified Delphi process to gain consensus on the use of QW-HFD in future trials of acute respiratory failure. Eighty-four percent of respondents preferred the use of QW-HFD to HFD alone in this context. Participants felt that it was more patient-centered and better aligned with goals of patients, families, and clinicians. It recommended the EQ-5D as the HRQoL measure to adjust HFD. However, QW-HFD is hampered by being more resource intensive, requiring more frequent and longer follow-up; participants in this study favored at least quarterly follow-up for a minimum of 24 months.

### Readmissions

The majority of studies did include readmissions to hospital in their calculation of HFD. Hospital readmission is common in survivors of critical illness and is of importance to patients and healthcare services (26–28). However, classifying and defining readmissions is nuanced and influenced by acute illness necessitating the initial admission, preexisting comorbidities and/or frailty, and social circumstances (28). HFD can capture the number and duration of hospital readmissions but do not distinguish between “avoidable” and “unavoidable” readmissions or between “related” and “unrelated” admissions. Additionally, readmissions are less likely to be related to the intervention when longer follow-up durations are used (26). We also recognize that some readmissions are considered of value to patients and their families or caregivers (e.g., symptom relief from dyspnea).

### Follow-Up Duration

The most common duration of follow-up for HFD was 90 days. Auriemma et al (3) recommend a follow-up duration of at least 90 days, especially for populations with a prolonged baseline hospital length of stay. However, they acknowledged further work is required to consider the patient-centeredness and statistical power of HFD at different follow-up durations. The ANZICS Clinical Trials Group consensus panel also recommended a follow-up duration of 90 days, but this requires further validation (22). A key consideration is the intervention, in particular, the timing of administration/delivery and the expected effects. Longer follow-up time points may allow patient-centered benefits of any interventions to be captured and allow sufficient time to capture repeat ED visits and hospitalizations (29). This would minimize the risk of observing small differences in HFD owing only to changes in the index hospital length of stay, including those introduced by time spent awaiting post-acute care location placement (e.g., nursing home).

### Minimal Clinically Important Difference

We found that of the 12 studies that use HFD as a primary outcome, three have not specified an MCID (30–32). Nine studies have specified an MCID, but three of these have not referenced for their choice of MCID (although one of these is a Bayesian secondary analysis of an included study) (33–35). Five have referenced pilot trials or cohort studies, which potentially suggests that they have used a distribution-based method for calculating the MCID. Ideally, the MCID would be derived using a method that involves consultation with relevant stakeholders. For this reason, distribution-based methods are not recommended in isolation (17).

In a two-part survey of 79 stakeholders, the most common reported MCID was 8–14 days and NIM was 1–2 days when a range of values was offered (13). However, this study used a hypothetical trial with a baseline of 120 HFD in a 6 month follow-up period, which is far longer than most of the studies included in our review. The protocol for one of the completed studies included, Early and Sustained Lactobacillus plantarum Probiotic Therapy in Critical Illness: The Randomised, Placebo-Controlled, Restoration of Gut Microflora in Critical Illness Trial (ROCIT), which refers to a “specially convened forum of consumers including ICU survivors and next-of-kin (Consumer and Community Health Research Network, University of Western Australia, WA)” who considered a difference of 4 days meaningful for HFD with a follow-up duration of 60 days (36).

A pilot RCT of iron and erythropoietin after critical illness (Iron and Erythropoietin to Heal and Recover after Intensive Care [ITHRIVE]) asked all pilot participants to consider the minimum number of additional days at home until day 90 that would be of value to them. It reported this as a secondary outcome, with a mean of 3 days (37). Similarly, in a planned RCT comparing IV iron and placebo in patients undergoing major surgery with HFD at 30 days as its primary outcome, an MCID of 2 days was chosen (38). This highlights how MCID will vary between different clinical populations and is influenced by the baseline mortality and hospital length of stay.

### Statistical Analysis

The most common statistical analysis approach was to use nonparametric tests. A number of other studies used a regression based approach. Often, the heavily skewed and bimodal distribution of HFD (an inflated number of both 0 and maximum values) requires careful consideration. In their review of days alive without life support and other count outcomes, Granholm et al (8) compared four specific types of regression models of increasing complexity (linear, hurdle-negative binomial, zero-one inflated beta, and cumulative logistic regression) in a reanalysis of the Effect of 12 mg vs 6 mg of Dexamethasone on the Number of Days Alive Without Life Support in Adults With COVID-19 and Severe Hypoxemia (COVID-STEROID 2) trial. They used models rather than simpler nonparametric tests as they allow for comparison of more than two treatment arms, adjustment for multiple covariates, quantification of effect measures, and their ability to generate posterior distributions with prior information for Bayesian analysis. They found there was no universal best model; simpler models were able to estimate the group mean and were simpler to interpret, whereas more complex models provided a better fit to data but came with greater uncertainty.

### Strengths and Limitations

Our review has strengths. We followed PRISMA recommendations, and our protocol was prospectively registered. We also performed a comprehensive search and used duplicate data extraction and risk of bias assessments. This review also has limitations. The construct captured by HFD is identified by an evolving number of terms, although the search was run iteratively to capture additional terms identified in the search, it is possible that studies were missed. We also restricted our search to English-only publications, which may have missed additional studies. Additionally, the single-author data extraction method used is more prone to bias and transcription errors.

## CONCLUSIONS

HFD is a feasible, objective, and patient-centered outcome measure that is increasingly being used in critical care research. This review, which included 110 studies, has highlighted substantial variation in definition, calculation, and analysis of HFD. We make a number of recommendations for the use of HFD and highlight avenues for future research to ensure optimal use of this outcome.

## ACKNOWLEDGMENTS

We thank Marshall Dozier, MA, PgDipLIS, EdD, FHEA, College Lead for Library Academic Support (College of Medicine and Veterinary Medicine) and Academic Support Librarian (Medicine), University of Edinburgh, for her invaluable help in creating the search strategy for this review. Also, we thank Edward Litton, MBChB, MSc, FCICM, Associate Professor, University of Western Australia Medical School, for his support with the review. We are grateful to those authors who replied to requests for more information on their studies; a full list of whom is included in **Supplementary Material 3** (https://links.lww.com/CCM/H794).

## Supplementary Material
