# Supplementary material for: Reporting of Hospital-Free Days As an Outcome Measure in Critical Care Trials: A Systematic Review
Source: Crit Care Med. 2025 Sep 24;53(12):e2686–97. doi: 10.1097/CCM.0000000000006858 (PMC12655879; doi:10.1097/CCM.0000000000006858)
Supplement: Supplementary file 1 [file ccm-53-e2686-s001.pdf]

Supplemental Digital Content

Supplementary Material 1. Search strategy
Supplementary Material 2. Sample data extraction form
Supplementary Material 3. List of authors who kindly replied to requests for more information
Supplementary Table S1. Completed PRISMA 2020 checklist
Supplementary Table S2. Table of all included studies
Supplementary Table S3. Table of risk of bias in included studies
Supplementary Table S4. Table of all included studies with published results
Supplementary Table S5. Table of frequencies of variations of HFD outcome name in included studies
Supplementary Table S6. Table of included studies with HFD as a primary outcome showing features of the definition of HFD
Supplementary Table S7. Table of recommendations for the use of HFD in critical care research (with justification)

Supplementary Material 1. Search strategy

CENTRAL (n=595)

#1 (intensive* OR critical* OR neurointensive* OR neurocritical* OR coronary) near/3 (care OR therap* OR treatment*)
#2 (critical* OR serious* OR severe*) NEAR/2 (ill* OR unwell OR injur* OR wound*)
#3 ((critical* OR intensive* OR trauma*)):so
#4 "high dependency"
#5 ICU OR MICU OR CICU OR CVICU OR CCU OR SICU OR POCCU OR ITU OR HDU
#6 {OR #1-#5}
#7 "hospital free days"
#8 ("days out of" near/3 hospital)
#9 "days alive and out of the ICU and hospital"
#10 "number of alive" near/3 days
#11 days near/5 alive near/5 hospital
#12 "days" near/3 "at home"
#13 HFD OR HFDs OR DAOH OR DAH OR DAAH
#14 {OR #7-#13}
#15 #6 AND #14 in Trial

Embase (n=792)

1. exp randomized controlled trial/
2. Controlled clinical trial/
3. random$.ti,ab.
4. randomization/
5. intermethod comparison/
6. placebo.ti,ab.
7. (compare OR compared OR comparison).ti.
8. ((evaluated OR evaluate OR evaluating OR assessed OR assess) and (compare OR compared OR comparing OR comparison)).ab.
9. (open adj label).ti,ab.
10. ((double OR single OR doubly OR singly) adj (blind OR blinded OR blindly)).ti,ab.
11. double blind procedure/
12. parallel group$1.ti,ab.
13. (crossover OR cross over).ti,ab.
14. ((assign$ OR match OR matched OR allocation) adj5 (alternate OR group$1 OR intervention$1 OR patient$1 OR subject$1 OR participant$1)).ti,ab.
15. (assigned OR allocated).ti,ab.
16. (controlled adj7 (study OR design OR trial)).ti,ab.
17. (volunteer OR volunteers).ti,ab.
18. human experiment/
19. trial.ti.
20. or/1-19
21. (random$ adj sampl$ adj7 ("cross section$" OR questionnaire$1 OR survey$ OR database$1)).ti,ab. not (comparative study/ OR controlled study/ OR randomi?ed controlled.ti,ab. OR randomly assigned.ti,ab.)
22. Cross-sectional study/ not (exp randomized controlled trial/ OR controlled clinical study/ OR controlled study/ OR randomi?ed controlled.ti,ab. OR control group$1.ti,ab.)
23. (((case adj control$) and random$) not randomi?ed controlled).ti,ab.
24. Systematic review.ti,ab. not (trial OR study).ti.
25. (nonrandom$ not random$).ti,ab.
26. "random field$".ti,ab.
27. (random cluster adj3 sampl$).ti,ab.
28. (review.ab. and review.pt.) not trial.ti.
29. "we searched".ab. and (review.ti. OR review.pt.)
30. "update review".ab.
31. (databases adj4 searched).ab.
32. (rat OR rats OR mouse OR mice OR swine OR porcine OR murine OR sheep OR lambs OR pigs OR piglets OR rabbit OR rabbits OR cat OR cats OR dog OR dogs OR cattle OR bovine OR monkey OR monkeys OR trout OR marmoset$1).ti. and animal experiment/
33. Animal experiment/ not (human experiment/ OR human/)
34. or/21-33
35. 20 not 34
36. exp Intensive Care/
37. Intensive Care Unit/
38. Coronary Care Unit/
39. Critical Illness/
40. Critically Ill Patient/
41. ((intensive* OR critical* OR neurointensive* OR neurocritical* OR coronary) adj3 (care OR therap* OR treatment*)).tw.

42. ((critical* OR serious* OR severe*) adj2 (ill* OR unwell OR injur* OR wound*)).tw.
43. (critical* OR intensive* OR trauma*).jn.
44. "high dependency".tw.
45. (ICU OR MICU OR CICU OR CVICU OR CCU OR SICU OR POCCU OR ITU OR HDU).tw.
46. 36 OR 37 OR 38 OR 39 OR 40 OR 41 OR 42 OR 43 OR 44 OR 45
47. "hospital free days".mp.
48. ("days out of" adj3 hospital).mp.
49. "days alive and out of the ICU and hospital".mp.
50. ("number of alive" adj3 days).tw.
51. (days adj5 alive adj5 hospital).mp.
52. (days adj3 "at home").mp.
53. (HFD OR HFDs OR DAOH OR DAH OR DAAH).tw.
54. 47 OR 48 OR 49 OR 50 OR 51 OR 52 OR 53
55. 35 and 46 and 54

MEDLINE (n=379)

1. exp randomized controlled trial/
2. controlled clinical trial.pt.
3. randomized.ab.
4. placebo.ab.
5. drug therapy.fs.
6. randomly.ab.
7. trial.ab.
8. groups.ab.
9. or/1-8
10. exp Intensive Care Units/
11. exp Critical Care/
12. Critical Illness/
13. exp Perioperative Care/
14. Critical Care Nursing/
15. Trauma Centers/
16. ((intensive* OR critical* OR neurointensive* OR neurocritical* OR coronary) adj3 (care OR therap* OR treatment*)).tw.
17. ((critical* OR serious* OR severe*) adj2 (ill* OR unwell OR injur* OR wound*)).tw.
18. (critical* OR intensive* OR trauma*).jn.
19. "high dependency".tw.
20. (ICU OR MICU OR CICU OR CVICU OR CCU OR SICU OR POCCU OR ITU OR HDU).tw.
21. 10 OR 11 OR 12 OR 13 OR 14 OR 15 OR 16 OR 17 OR 18 OR 19 OR 20 666248
22. "hospital free days".mp.
23. ("days out of" adj3 hospital).mp.
24. "days alive and out of the ICU and hospital".mp.
25. ("number of alive" adj3 days).tw.
26. (days adj5 alive adj5 hospital).mp.
27. (days adj3 "at home").mp.
28. (HFD OR HFDs OR DAOH OR DAH OR DAAH).tw.
29. 22 OR 23 OR 24 OR 25 OR 26 OR 27 OR 28
30. 9 and 21 and 29

CINAHL Plus (n=278)

S1 MH randomized controlled trials
S2 MH double-blind studies
S3 MH single-blind studies
S4 MH random assignment
S5 MH pretest-posttest design
S6 MH cluster sample
S7 TI (randomised OR randomized)
S8 AB (random*)
S9 TI (trial)
S10 MH (sample size) AND AB (assigned OR allocated OR control)
S11 MH (placebos)
S12 PT (randomized controlled trial)
S13 AB (control W5 group)
S14 MH (crossover design) OR MH (comparative studies)
S15 AB (cluster W3 RCT)
S16 MH animals+
S17 MH (animal studies)
S18 TI (animal model*)
S19 S16 OR S17 OR S18
S20 MH (human)
S21 S19 NOT S20
S22 S1 OR S2 OR S3 OR S4 OR S5 OR S6 OR S7 OR S8 OR S9 OR S10 OR S11 OR S12 OR S13 OR S14 OR S15
S23 S22 NOT S21
S24 (MH "Critical Care+")
S25 (MH "Intensive Care Units+")
S26 (MH "Critical Illness")
S27 (MH "Catastrophic Illness")
S28 (MH "Critically Ill Patients")
S29 (MH "Critical Care Nursing+")
S30 (MH "Trauma Centers")
S31 TX ((intensive* OR critical* OR neurointensive* OR neurocritical* OR coronary) N3 (care OR therap* OR treatment*))
S32 TX ((critical* OR serious* OR severe*) N2 (ill* OR unwell OR injur* OR wound*))
S33 JN (critical* OR intensive* OR trauma*)
S34 TX "high dependency"
S35 TX (ICU OR MICU OR CICU OR CVICU OR CCU OR SICU OR POCCU OR ITU OR HDU)
S36 S24 OR S25 OR S26 OR S27 OR S28 OR S29 OR S30 OR S31 OR S32 OR S33 OR S34 OR S35
S37 TX ("hospital free days")
S38 TX ("days out of" w3 hospital)
S39 TX ("days alive and out of the ICU and hospital")
S40 TX ("number of alive" n3 days)
S41 TX (days n5 alive n5 hospital)
S42 TX ("days" n3 "at home")
S43 TX (HFD OR HFDs OR DAOH OR DAH OR DAAH)
S44 S37 OR S38 OR S39 OR S40 OR S41 OR S42 OR S43
S45 S23 AND S36 AND S44

Clinicaltrials.gov (n=89)

[Filter] interventional
[Other terms]
("intensive care" OR "critical care" OR "neurointensive care" OR "neurocritical care" OR "coronary care" OR "intensive therapy" OR "critical therapy" OR "neurointensive therapy" OR "neurocritical therapy" OR "coronary therapy" OR "intensive therapies" OR "critical therapies" OR "neurointensive therapies" OR "neurocritical therapies" OR "coronary therapies" OR "intensive treatment" OR "critical treatment" OR "neurointensive treatment" OR "neurocritical treatment" OR "coronary treatment" OR "critical illness" OR "critical injury" OR "critically ill" OR "critically unwell" OR "critically injured" OR "critically wounded" OR "serious illness" OR "serious injury" OR "seriously ill" OR "seriously unwell" OR "seriously injured" OR "seriously wounded" OR "severe illness" OR "severe injury" OR "severely ill" OR "severely unwell" OR "seriously injured" OR "severely wounded" OR "high dependency" OR ICU OR MICU OR CICU OR CVICU OR CCU OR SICU OR POCCU OR ITU OR HDU) AND ("hospital free day" OR "hospital free days" OR "days out of hospital" OR "days out of the hospital" OR "days alive and out of hospital" OR "days alive and out of the hospital" OR "days alive and out of the ICU and hospital" OR "days at home" OR "days spent at home" OR "days alive and at home" OR "days alive at home" OR "number of alive days" OR HFD OR HFDs OR DAOH OR DAH OR DAAH)

ISRCTN (n=1)

[Text search]
(intensive OR critical OR neurocritical OR neurointensive OR "coronary care" OR critically OR "serious illness" OR "serious injury" OR "seriously ill" OR "seriously unwell" OR "seriously injured" OR "severe illness" OR "severe injury" OR "severely ill" OR "severely unwell" OR "seriously injured" OR "high dependency" OR ICU OR MICU OR CICU OR CVICU OR CCU OR SICU OR POCCU OR ITU OR HDU) AND ("hospital free day" OR "hospital free days" OR "days out of hospital" OR "days out of the hospital" OR "days alive and out of hospital" OR "days alive and out of the hospital" OR "days alive and out of the ICU and hospital" OR "days at home" OR "days spent at home" OR "days alive and at home" OR "days alive at home" OR "number of alive days" OR HFD OR HFDs OR DAOH OR DAH OR DAAH)

Supplementary Material 2. Sample data extraction form

General Information

Title
Free text

Study acronym
Free text

Type of study:
Trial registry
Published protocol or statistical plan
Published manuscript with results
Secondary analysis
Other (*free text*)

NCT number
Free text

ACTRN or EUCTR or DRKS or ISRCTN
Free text

Status:
Not yet recruiting
Recruiting
Withdrawn
Completed
Unknown
Other (*free text*)

Lead author contact details
Other (*free text*)

Lead author title, first and last name
Other (*free text*)

Continent in which study was conducted
North America
South America
Europe
Asia
Australasia
Africa

Notes
Other (*free text*)

Methods

Aim of study
Free text

Design
Single centre RCT
Multi centre RCT
Cluster RCT

Recruitment start date (mm/yyyy)
Free text

Recruitment end date (mm/yyyy)
Free text

Length of maximum follow up
Free text

Participants

Population description
Free text

List inclusion criteria
Free text

List exclusion criteria
Free text

Method of follow up
Questionnaires
Routinely collected healthcare records (local)
Routinely collected healthcare records (national)
Telephone
Not specified
Other

Total number of participants screened
Free text

Total number of participants included
Free text

Number of patients with HFD outcome available

Free text

Reasons given for loss to follow up for HFD and number of patients lost
No reason given
Death
Withdrawn consent
Didn't return questionnaire
Couldn't contact by telephone
Other

Intervention and comparison

Intervention
Free text

Comparison
Free text

Duration of treatment period
Free text

Outcomes

Primary outcome
HFD
Mortality
Hospital length of stay
Readmission
Other (*free text*)

Primary outcome time point if mortality/ other but not HFD/ hospital length of stay/ readmission
7 days
14 days
28 days
30 days
60 days
90 days
Other (*free text*)

Outcomes included
HFD
Mortality
Hospital length of stay
Readmission

HFD outcome

Primary outcome
Secondary outcome
Other (*free text*)

HFD outcome name
Free text

HFD outcome description
Free text

Resources searched for HFD outcome description
Trial registry
Protocol
Statistical analysis plan
Manuscript
Supplementary material
Other (*free text*)

HFD outcome time point
7 days
14 days
28 days
30 days
60 days
90 days
Other (*free text*)

Mortality outcome time point
7 days
14 days
28 days
30 days
60 days
90 days
ICU
Hospital
Other (*free text*)

Readmission outcome description
Free text

Readmission outcome time point
7 days
14 days
28 days
30 days
60 days

90 days
Other (*free text*)

Statistical methods

Minimally clinically important difference (MCID) if HFD is the primary outcome
Free text

Reference for MCID for HFD outcome
Free text

Statistical methods by outcome
HFD outcome
Mortality outcome
Hospital length of stay outcome
Readmission outcome

Were stratification variables used for the HFD outcome?
Yes
No
Not specified

If yes, list the stratification variables used for the HFD outcome
Free text

Results

Primary outcome results if not HFD/ mortality/ hospital length of stay/ readmission
Free text

HFD outcome results
Free text

Mortality outcome results
Free text

Hospital length of stay outcome results
Free text

Readmission outcome results
Free text

Discussion and conclusion

Key points made about HFD outcome used
Free text

Key conclusions of study
Free text

References to other relevant studies regarding HFD outcomes
Free text

Risk of Bias Assessment

Blinding of outcome assessment
High
Low
Unsure

Incomplete outcome data
High
Low
Unsure

Selective reporting
High
Low
Unsure

Supplementary Material 3. List of authors who kindly replied to requests for more information

Nina Andersen-Ranberg
Julia Bels
Peter Bentzer
Morten Bestle
Roy Brower
Anthony Delaney
Ra'eesa Doola
Edward Fysh
Adit Ginde
Ewan Goligher
Matthieu Jabaudon-Gandet
David Leaf
Edward Litton
Nick Meier
Mark Metersky
Stan Miele
Jacob Møller
John Myburgh
Ary Neto
Marlies Ostermann
Brandon Oto
Paul Palevsky
Sandra Peake
Ithan Peltan
Anders Perner
Ville Pettil
Alex Poole
Annette Reboli
Antoine Roquilly
Matthew Semler
Bodil Steen Rasmussen
Boyd Taylor Thompson
Sophie Toya
Sine Wichmann
Paul Young
Ryan Zarychanski

Supplementary Table S1. Completed PRISMA 2020 checklist

Section and Topic	Item #	Checklist item	Location where item is reported
**TITLE**			
Title	1	Identify the report as a systematic review.	1
**ABSTRACT**			
Abstract	2	See the PRISMA 2020 for Abstracts checklist.	2
**INTRODUCTION**			
Rationale	3	Describe the rationale for the review in the context of existing knowledge.	5
Objectives	4	Provide an explicit statement of the objective(s) or question(s) the review addresses.	5
**METHODS**			
Eligibility criteria	5	Specify the inclusion and exclusion criteria for the review and how studies were grouped for the syntheses.	7
Information sources	6	Specify all databases, registers, websites, organisations, reference lists and other sources searched or consulted to identify studies. Specify the date when each source was last searched or consulted.	7
Search strategy	7	Present the full search strategies for all databases, registers and websites, including any filters and limits used.	7
Selection process	8	Specify the methods used to decide whether a study met the inclusion criteria of the review, including how many reviewers screened each record and each report retrieved, whether they worked independently, and if applicable, details of automation tools used in the process.	7
Data collection process	9	Specify the methods used to collect data from reports, including how many reviewers collected data from each report, whether they worked independently, any processes for obtaining or confirming data from study investigators, and if applicable, details of automation tools used in the process.	7
Data items	10a	List and define all outcomes for which data were sought. Specify whether all results that were compatible with each outcome domain in each study were sought (e.g. for all measures, time points, analyses), and if not, the methods used to decide which results to collect.	Supplementary material
	10b	List and define all other variables for which data were sought (e.g. participant and intervention characteristics, funding sources). Describe any assumptions made about any missing or unclear information.	Supplementary material
Study risk of bias assessment	11	Specify the methods used to assess risk of bias in the included studies, including details of the tool(s) used, how many reviewers assessed each study and whether they worked independently, and if applicable, details of automation tools used in the process.	8
Effect measures	12	Specify for each outcome the effect measure(s) (e.g. risk ratio, mean difference) used in the synthesis or presentation of results.	N/A
Synthesis methods	13a	Describe the processes used to decide which studies were eligible for each synthesis (e.g. tabulating the study intervention characteristics and comparing against the planned groups for each synthesis (item #5)).	8
	13b	Describe any methods required to prepare the data for presentation or synthesis, such as handling of missing summary statistics, or data conversions.	8

Section and Topic	Item #	Checklist item	Location where item is reported
	13c	Describe any methods used to tabulate or visually display results of individual studies and syntheses.	N/A
	13d	Describe any methods used to synthesize results and provide a rationale for the choice(s). If meta-analysis was performed, describe the model(s), method(s) to identify the presence and extent of statistical heterogeneity, and software package(s) used.	8
	13e	Describe any methods used to explore possible causes of heterogeneity among study results (e.g. subgroup analysis, meta-regression).	N/A
	13f	Describe any sensitivity analyses conducted to assess robustness of the synthesized results.	N/A
Reporting bias assessment	14	Describe any methods used to assess risk of bias due to missing results in a synthesis (arising from reporting biases).	8
Certainty assessment	15	Describe any methods used to assess certainty (or confidence) in the body of evidence for an outcome.	8
**RESULTS**			
Study selection	16a	Describe the results of the search and selection process, from the number of records identified in the search to the number of studies included in the review, ideally using a flow diagram.	Supplementary material
	16b	Cite studies that might appear to meet the inclusion criteria, but which were excluded, and explain why they were excluded.	Supplementary material
Study characteristics	17	Cite each included study and present its characteristics.	Supplementary material
Risk of bias in studies	18	Present assessments of risk of bias for each included study.	Supplementary material
Results of individual studies	19	For all outcomes, present, for each study: (a) summary statistics for each group (where appropriate) and (b) an effect estimate and its precision (e.g. confidence/credible interval), ideally using structured tables or plots.	Supplementary material
Results of syntheses	20a	For each synthesis, briefly summarise the characteristics and risk of bias among contributing studies.	N/A
	20b	Present results of all statistical syntheses conducted. If meta-analysis was done, present for each the summary estimate and its precision (e.g. confidence/credible interval) and measures of statistical heterogeneity. If comparing groups, describe the direction of the effect.	N/A
	20c	Present results of all investigations of possible causes of heterogeneity among study results.	N/A
	20d	Present results of all sensitivity analyses conducted to assess the robustness of the synthesized results.	N/A
Reporting biases	21	Present assessments of risk of bias due to missing results (arising from reporting biases) for each synthesis assessed.	N/A
Certainty of evidence	22	Present assessments of certainty (or confidence) in the body of evidence for each outcome assessed.	N/A
**DISCUSSION**			

Section and Topic	Item #	Checklist item	Location where item is reported
Discussion	23a	Provide a general interpretation of the results in the context of other evidence.	13
	23b	Discuss any limitations of the evidence included in the review.	20
	23c	Discuss any limitations of the review processes used.	20
	23d	Discuss implications of the results for practice, policy, and future research.	Table 3
**OTHER INFORMATION**			
Registration and protocol	24a	Provide registration information for the review, including register name and registration number, or state that the review was not registered.	7
	24b	Indicate where the review protocol can be accessed, or state that a protocol was not prepared.	7
	24c	Describe and explain any amendments to information provided at registration or in the protocol.	7
Support	25	Describe sources of financial or non-financial support for the review, and the role of the funders or sponsors in the review.	1
Competing interests	26	Declare any competing interests of review authors.	1
Availability of data, code and other materials	27	Report which of the following are publicly available and where they can be found: template data collection forms; data extracted from included studies; data used for all analyses; analytic code; any other materials used in the review.	1, Supplementary material

From: Page MJ, McKenzie JE, Bossuyt PM, Boutron I, Hoffmann TC, Mulrow CD, et al. The PRISMA 2020 statement: an updated guideline for reporting systematic reviews. BMJ 2021;372:n71. doi: 10.1136/bmj.n71

Supplementary Table S2. Table of all included studies

Title	Type of Study	Trial Registration Number	HFD Outcome
Clinical outcomes of empirical high-dose meropenem in critically ill patients with sepsis and septic shock: a randomized controlled trial	Published manuscript with results	NCT03344627	Secondary
Lower vs Higher Oxygenation Target and Days Alive Without Life Support in COVID-19	Published manuscript with results	NCT04425031	Secondary
Intensity of Renal Support in Critically Ill Patients with Acute Kidney Injury	Published manuscript with results	NCT00076219	Secondary
Results of the CONTROL Trial: Efficacy and Safety of Recombinant Activated Factor VII in the Management of Refractory Traumatic Hemorrhage	Published manuscript with results	NCT00184548<br>NCT00323570 (merged)	Secondary
Randomized trial of initial trophic versus full-energy enteral nutrition in mechanically ventilated patients with acute respiratory failure	Published manuscript with results	NCT00252616	Secondary
Etomidate versus ketamine for rapid sequence intubation in acutely ill patients: a multicentre randomised controlled trial	Published manuscript with results	NCT00440102	Secondary
Hydroxyethyl Starch 130/0.42 versus Ringer,Äôs Acetate in Severe Sepsis	Published manuscript with results	NCT00962156	Secondary
Enteral nutrition with eicosapentaenoic acid, g-linolenic acid and antioxidants in the early treatment of sepsis: results from a multicenter, prospective, randomized, double-blinded, controlled study: the INTERSEPT Study	Published manuscript with results	NCT00981877	Secondary
APCAP - activated protein C in acute pancreatitis: a double-blind randomized human pilot trial	Published manuscript with results	NCT01017107	Secondary
A Randomized Trial of an Intensive Physical Therapy Program for Patients with Acute Respiratory Failure	Published manuscript with results	NCT01058421	Secondary
Effect of Ganciclovir on IL-6 Levels Among Cytomegalovirus-Seropositive Adults With Critical Illness	Published manuscript with results	NCT01335932	Secondary
Lower versus Higher Hemoglobin Threshold for Transfusion in Septic Shock	Published manuscript with results	NCT01485315	Secondary
Transfusion of Plasma, Platelets, and Red Blood Cells in a 1:1:1 vs a 1:1:2 Ratio and Mortality in Patients With Severe Trauma	Published manuscript with results	NCT01545232	Secondary
Effect of Vitamin C Infusion on Organ Failure and Biomarkers of Inflammation and Vascular Injury in Patients With Sepsis and Severe Acute Respiratory Failure	Published manuscript with results	NCT02106975	Secondary
Immunoglobulin G for patients with necrotising soft tissue infection (INSTINCT): a randomised, blinded, placebo-controlled trial	Published manuscript with results	NCT02111161	Secondary
Energy-Dense versus Routine Enteral Nutrition in the Critically Ill	Published manuscript with results	NCT02306746	Secondary
Effect of Selective Decontamination of the Digestive Tract on Hospital Mortality in Critically Ill Patients<br>Receiving Mechanical Ventilation	Published manuscript with results	NCT02389036	Secondary
Forced fluid removal in intensive care patients with acute<br>kidney injury: The randomised FFAKI feasibility trial	Published manuscript with results	NCT02458157	Secondary
Early Neuromuscular Blockade in the Acute Respiratory Distress Syndrome	Published manuscript with results	NCT02509078	Secondary
Timing of Initiation of Renal-Replacement Therapy in Acute Kidney Injury	Published manuscript with results	NCT02568722	Secondary
Therapeutic Hyperthermia Is Associated<br>With Improved Survival in Afebrile Critically Ill Patients With Sepsis: A Pilot Randomized Trial	Published manuscript with results	NCT02706275	Secondary
Early Active Mobilization during Mechanical Ventilation in the ICU	Published manuscript with results	NCT03133377	Primary
Lower or Higher Oxygenation Targets for Acute Hypoxemic Respiratory Failure	Published manuscript with results	NCT03174002	Secondary

Haloperidol for the Treatment of Delirium in ICU Patients	Published manuscript with results	NCT03392376	Primary
Early Restrictive or Liberal Fluid Management for Sepsis-Induced Hypotension	Published manuscript with results	NCT03434028	Secondary
Oxygen-Saturation Targets for Critically Ill Adults Receiving Mechanical Ventilation	Published manuscript with results	NCT03537937	Secondary
Restriction of Intravenous Fluid in ICU Patients with Septic Shock	Published manuscript with results	NCT03668236	Secondary
Goal-directed fluid removal with furosemide versus placebo in intensive care patients with fluid overload: A randomised, blinded trial (GODIF trial,ÄîFirst version)	Published manuscript with results	NCT04180397	Primary
Randomized Phase 3 Trial of Ruxolitinib for COVID-19-Associated Acute Respiratory Distress Syndrome	Published manuscript with results	NCT04377620	Secondary
Evaluation of the Efficacy of Nicotine Patches in SARS-CoV2 (COVID-19) Infection in Intensive Care Unit Patients	Published manuscript with results	NCT04598594	Secondary
Interferon gamma-1b for the prevention of hospital-acquired pneumonia in critically ill patients: a phase 2, placebo-controlled randomized clinical trial	Published manuscript with results	NCT04793568	Secondary
Effects of gabapentin on slow-wave sleep period in critically ill adult patients: a randomized controlled trial	Published manuscript with results	NCT04818450	Secondary
Intravenous aviptadil and remdesivir for treatment of<br>COVID-19-associated hypoxaemic respiratory failure in<br>the USA (TESICO): a randomised, placebo-controlled trial	Published manuscript with results	NCT04843761	Secondary
Early and sustained Lactobacillus plantarum probiotic therapy in critical illness: the randomised, placebo-controlled, restoration of gut microflora in critical illness trial (ROCIT)	Published manuscript with results	ACTRN12617000783325	Primary
Iron and erythropoietin to heal and recover after intensive care (ITHRIVE): A pilot randomised clinical trial	Published manuscript with results	ACTRN12621000595819	Primary
The Effect of a Liberal Approach to Glucose Control in Critically Ill<br>Patients with Type 2 Diabetes	Published manuscript with results	ACTRN 12616001135404	Other: Secondary in trial registry and protocol, variation in published manuscript used looking at discharge destination
A Phase II Cluster-Crossover Randomized Trial of Fentanyl versus Morphine for Analgosedation in Mechanically Ventilated Patients	Published manuscript with results	ACTRN12619000939190	Secondary
Acetaminophen for Fever in Critically Ill Patients with Suspected Infection	Published manuscript with results	ACTRN12611000981921<br>ACTRN12612000513819	Secondary
Remi-fent 1 - A pragmatic randomised controlled study to evaluate the feasibility of using remifentanil or fentanyl as sedation adjuncts in mechanically ventilated patients	Published manuscript with results	ACTRN12620000719932	Secondary
Adjunctive Glucocorticoid Therapy in Patients with Septic Shock	Published manuscript with results (primary outcome results from poster presentation)	NCT01448109	Secondary
Rationale and design of DanGer shock: Danish-German cardiogenic shock trial	Published protocol or statistical plan	NCT01633502	Secondary
Interactions in clinical trials: Protocol and statistical analysis plan for an explorative study of four randomized ICU trials on use of pantoprazole, oxygenation targets, haloperidol and intravenous fluids	Published protocol or statistical plan	NCT02467621 (SUP-ICU)<br>NCT03174002 (HOT-ICU)<br>NCT03392376 (AID-ICU)<br>NCT03668236 (CLASSIC)	Secondary

Targeted tissue perfusion versus macrocirculation-guided standard care in patients with septic shock (TARTARE-2S): study protocol and statistical analysis plan for a randomized controlled trial	Published protocol or statistical plan	NCT02579525	Secondary
Prognosticating Outcomes and Nudging Decisions with Electronic Records in the Intensive Care Unit Trial Protocol	Published protocol or statistical plan	NCT03139838	Secondary
Awake prone positioning in nonintubated spontaneous breathing ICU patients with acute hypoxemic respiratory failure (PRONELIFE),Äîprotocol for a randomized clinical trial	Published protocol or statistical plan	NCT04142736	Secondary
Furosemide versus placebo for fluid overload in intensive care patients,ÄîThe randomised GODIF trial second version: Statistical analysis plan	Published protocol or statistical plan	NCT04180397	Primary
Adjunctive IgM-enriched immunoglobulin therapy with a personalised dose based on serum IgM-titres versus standard dose in the treatment of septic shock: a randomised controlled trial (IgM-fat trial)	Published protocol or statistical plan	NCT04182737	Secondary
Design and Rationale of the Sevoflurane for Sedation in Acute Respiratory Distress Syndrome (SESAR) Randomized Controlled Trial	Published protocol or statistical plan	NCT04235608	Secondary
Nitric Oxide Gas Inhalation in Severe Acute Respiratory Syndrome in COVID-19	Published protocol or statistical plan	NCT04306393	Secondary
Replacing protein via enteral nutrition in a stepwise approach in critically ill patients: the REPLENISH randomized clinical trial protocol	Published protocol or statistical plan	NCT04475666	Secondary
A multicenter cluster randomized, stepped wedge implementation trial for targeted normoxia in critically ill trauma patients: study protocol and statistical analysis plan for the Strategy to Avoid Excessive Oxygen (SAVE-O2) trial	Published protocol or statistical plan	NCT04534959	Secondary
PRotEin Provision in Critical IllneSs (PRECISe)	Published protocol or statistical plan	NCT04633421	Secondary
Dapagliflozin in patients with critical illness: rationale<br>and design of the DEFENDER study	Published protocol or statistical plan	NCT05558098	Secondary
Protocol and statistical analysis plan for the Mode of Ventilation During Critical IllnEss (MODE) trial	Published protocol or statistical plan	NCT05563779	Secondary
Effectiveness of the ABCDEF bundle on delirium, functional outcomes and quality of life in intensive care patients: a study protocol for a randomised controlled trial with embedded process evaluation	Published protocol or statistical plan	ACTRN12620000736943	Secondary
Efficacy and safety outcomes of drainage of intensive care pleural effusions<br>Study protocol for the ESODICE randomized controlled trial	Published protocol or statistical plan	ACTRN12620000519954	Secondary
Study protocol for TARGET protein: The effect of augmented administration of enteral protein to critically ill adults on clinical outcomes: A cluster randomised, cross-sectional, double cross-over, clinical trial	Published protocol or statistical plan	ACTRN12621001484831	Primary
Comparison of empirical high-dose and low-dose of meropenem in critically ill patients with sepsis and septic shock	Published protocol or statistical plan	Research Registry 6023	Secondary
Randomised, controlled, feasibility trial comparing vasopressor infusion administered via peripheral cannula versus central venous catheter for critically ill adults: a study protocol	Published protocol or statistical plan	ACTRN12621000721808	Primary
Resource Utilization and Cost of Treatment with Anidulafungin or Fluconazole for Candidaemia and Other Forms of Invasive Candidiasis	Secondary analysis	NCT00056368	Secondary
Hydrocortisone plus fludrocortisone for community acquired pneumonia-related septic shock: a subgroup analysis of the APROCCHSS phase 3 randomised trial	Secondary analysis	NCT00625209	Secondary

Long-term outcomes in patients with severe sepsis randomised to resuscitation with hydroxyethyl starch 130/0.42 or Ringer‚Äôs acetate	Secondary analysis	NCT00962156	Secondary
One-Year Outcomes in Patients With Acute Respiratory Distress Syndrome Enrolled in a Randomized Clinical Trial of Helmet Versus Facemask Noninvasive Ventilation	Secondary analysis	NCT01680783	Secondary
Selective digestive tract decontamination in critically ill adults with acute brain injuries: a post hoc analysis of a randomized clinical trial	Secondary analysis	NCT02389036	Secondary
A Bayesian reanalysis of the Standard versus Accelerated Initiation of Renal-Replacement Therapy in Acute Kidney Injury (STARRT-AKI) trial	Secondary analysis	NCT02568722	Secondary
Oxygenation targets in ICU patients with COVID-19: A post hoc subgroup analysis of the HOT-ICU trial	Secondary analysis	NCT03174002	Secondary
Haloperidol vs. placebo for the treatment of delirium in ICU patients: a pre-planned, secondary Bayesian analysis of the AID‚ÄêICU trial	Secondary analysis	NCT03392376	Primary
Outcome after prolonged sedation - IsoOut-Study	Secondary analysis	DRKS00020237	Secondary
Comparison of Two Methods of High Frequency Oscillatory Ventilation in Individuals With Acute Respiratory Distress Syndrome	Trial registry	NCT00399581	Secondary
A multi-center, randomized, double-blind, controlled dose-finding study to evaluate the safety and efficacy of MP4OX treatment plus standard of care in severely injured trauma patients with lactic acidosis due to hemorrhagic shock	Trial registry	NCT01004198	Secondary
Ticagrelor in Severe Community Acquired Pneumonia	Trial registry	NCT01998399	Secondary
Safety and Efficacy Study of Acthar in Subjects With ARDS	Trial registry	NCT02113735	Secondary
Safety Study of Inhaled Carbon Monoxide to Treat Sepsis-Induced Acute Respiratory Distress Syndrome (ARDS)	Trial registry	NCT02425579 (phase 1)<br>NCT03799874 (phase 2a)<br>NCT04870125 (phase 2b)	Secondary
Heparin Anticoagulation in Septic Shock	Trial registry	NCT03378466	Secondary
Acetaminophen and Ascorbate in Sepsis: Targeted Therapy to Enhance Recovery	Trial registry	NCT04291508	Secondary
Low dose of IL-2 In Acute respiratory DistrEss syndrome related to COVID-19	Trial registry	NCT04357444	Secondary
Accelerated Prone Position Ventilation of Patients With COVID-19	Trial registry	NCT04384900	Secondary
SedAting With Volatile Anesthetics Critically Ill COVID-19 Patients in ICU: Effects On Ventilatory Parameters And Survival	Trial registry	NCT04415060	Secondary
Coronavirus Induced Acute Kidney Injury: Prevention Using Urine Alkalinization	Trial registry	NCT04530448	Secondary
Plasma Exchange (PLEX) and Convalescent Plasma (CCP) in COVID-19 Patients With Multiorgan Failure	Trial registry	NCT04634422	Primary
Urine Alkalinisation to Prevent AKI in COVID-19	Trial registry	NCT04655716	Secondary
Transfusional Trigger in Post-operative Oncologic Patients in Critical Care	Trial registry	NCT04859855	Secondary
Oral Administration or Nasal Feeding of Huzhangxiefei Decoction for Treatment in Sepsis Induced Acute Lung Injury	Trial registry	NCT04940676	Secondary
Strategies for Anticoagulation During Venovenous ECMO	Trial registry	NCT04997265	Secondary
Early Antibiotics After Aspiration in ICU Patients	Trial registry	NCT05079620	Secondary
Helmet NIV vs. CPAP vs. High-flow Nasal Oxygen in Hypoxemic Respiratory Failure	Trial registry	NCT05089695	Secondary
Platform of Randomised Adaptive Clinical Trials in Critical Illness	Trial registry	NCT05440851	Secondary

Recovery From Acute Immune Failure in Septic Shock by Immune Cell Extracorporeal Therapy	Trial registry	NCT05442710	Secondary
Fibrinogen Early In Severe Trauma StudY II	Trial registry	NCT05449834	Primary
Proactive Prescription-based Fluid Management vs Usual Care in Critically Ill Patients on Kidney Replacement Therapy	Trial registry	NCT05473143	Secondary
The ECMO-Free Trial	Trial registry	NCT05486559	Secondary
Add-on Reparixin in Adult Patients With ARDS	Trial registry	NCT05496868	Secondary
Sedation, Temperature and Pressure After Cardiac Arrest and Resuscitation	Trial registry	NCT05564754	Secondary
SODium BICarbonate for Metabolic Acidosis in the ICU	Trial registry	NCT05697770	Secondary
Balanced Multi-Electrolyte Solution Versus Saline Trial for Diabetic KetoAcidosis	Trial registry	NCT05752279	Primary
The Danish Out-of-Hospital Cardiac Arrest Study	Trial registry	NCT05895838	Secondary
Baricitinib for Treating Hospital-acquired Pneumonia in Critically Ill Patients With a Proinflammatory Phenotype	Trial registry	NCT05914584	Secondary
Study of Safety and Efficacy of ALT-100mAb in Participants With Moderate/Severe ARDS	Trial registry	NCT05938036	Secondary
Protocolized Reduction of Non-resuscitation Fluids Versus Usual Care in Septic Shock Patients (REDUSE)	Trial registry	NCT06140147	Secondary
Empirical Meropenem Versus Piperacillin/Tazobactam for Adult Patients With Sepsis (EMPRESS)	Trial registry	NCT06184659	Secondary
Preemptive Treatment With Acyclovir in Intubated and Mechanically Ventilated Patients With Herpes (PTH2)	Trial registry	NCT06217406	Secondary
Vasopressin for Septic Shock Pragmatic Trial (VASSPR)	Trial registry	NCT06217562	Secondary
Dexamethasone for Treating Severe Hospital-acquired Pneumonia in Critically Ill Patients With a Proinflammatory Phenotype (HAP-DEX)	Trial registry	NCT06269900	Secondary
Multiple Study of Electroaccpuncture in ARDS	Trial registry	NCT06278675	Secondary
A prospective randomised, double-blind, placebo controlled pilot study of the effect of ketamine for adjunct analgesia therapy on opioid requirements in adult mechanically ventilated patients	Trial registry	ACTRN12622000920796	Secondary
REDuced CARBohydrate enteral formula compared to standard care to improve glycaemic control in critically ill tube fed patients ‚Äì a randomised controlled phase II trial	Trial registry	ACTRN12621000859886	Secondary
Evaluation of interventions linked to treatable traits in acute critical illness in adults to enable precision medicine: Data enabled Bayesian adaptive platform randomised clinical trial with embedded biological characterisation	Trial registry	ISRCTN82395639	Secondary
The Prophylaxis against Early Ventilator Associated Lower Respiratory Tract Infection (PREVENT LRTI) trial. A phase 2 multi-centre, randomized, double-blind, placebo-controlled parallel group clinical trial.	Trial registry	ACTRN12623000958684	Secondary
Clinical Outcome and Cost-effectiveness of Reduced Noradrenaline by Using a Lower Blood Pressure Target in Patients with Cardiogenic Shock from Acute Myocardial Infarction: A Multicenter Randomized Trial	Trial registry	EUCTR2021-005551-36-DK	Secondary
Activated Vitamin D for the Prevention and Treatment of Acute Kidney Injury	Trial registry	NCT02962102	Secondary

Supplementary Table S3. Table of risk of bias in included studies

**Domain**	**Number (%) of Studies**		
	Low Risk	High Risk	Uncertain Risk
Blinding of Outcome Assessment	65 (59.1)	23 (20.9)	22 (20.0)
Incomplete Outcome Data	44 (40)	3 (2.7)	63 (57.3)
Selective Reporting	36 (32.7)	11 (10.0)	63 (57.3)

Supplementary Table S4. Table of all included studies with published results

Title	Trial Registration Number	Population	Intervention	Comparison	Statistical Method for HFD Analysis	Primary Outcome	Primary Outcome Results
Clinical outcomes of empirical high-dose meropenem in critically ill patients with sepsis and septic shock: a randomized controlled trial	NCT03 344627	Adult patients admitted to ICU with sepsis and septic shock (n=76)	High dose meropenem 2g TDS	Standard dose meropenem 1g TDS	Mann-Whitney U test	Change in modified SOFA score at 4 days	No difference
Lower vs Higher Oxygenation Target and Days Alive Without Life Support in COVID-19	NCT04 425031	Adult patients admitted to ICU with COVID-19 receiving at least 10L/min of oxygen or mechanical ventilation (n=726)	PaO2 60mmgHg (lower oxygenation group)	PaO2 90mmHg (higher oxygenation group)	Van Elteren test	Days alive without life support at 90 days	Increase in lower oxygenation group
Intensity of Renal Support in Critically Ill Patients with Acute Kidney Injury	NCT00 076219	Critically ill adult patients with acute kidney injury (n=1124)	Intensive renal replacement therapy	Less-intensive renal replacement therapy	Analysis of variance	Mortality at 60 days	No difference
Results of the CONTROL Trial: Efficacy and Safety of Recombinant Activated Factor VII in the Management of Refractory Traumatic Haemorrhage	NCT00 184548 NCT00 323570 (merged)	Adults with blunt or penetrating trauma with ongoing bleeding after 4 units of red blood cells (n=573)	Recombinant factor VIIa	Placebo	Schoenfeld and Bernard method with analysis of covariance mode	Mortality at 30 days	No difference
Randomized trial of initial trophic versus full-energy enteral nutrition in mechanically ventilated patients with acute respiratory failure	NCT00 252616	Adult patients admitted to an ICU, mechanically ventilated, and enterally fed (n=200)	Initial trophic (10ml/h) enteral nutrition	Initial full energy enteral nutrition	Mann-Whitney U test	Ventilator-free days at 28 days	No difference
Etomidate versus ketamine for rapid sequence intubation in acutely ill patients: a multicentre randomised controlled trial	NCT00 440102	Acutely unwell adult patients requiring emergency intubation (n=655)	Etomidate 0.3mg/kg IV bolus	Ketamine 2mg/kg IV bolus	Not specified	Mean maximum SOFA score during first 3 days in ICU	No difference
Hydroxyethyl Starch 130/0.42 versus Ringer's Acetate in Severe Sepsis	NCT00 962156	Adult patients with severe sepsis admitted to ICU (n=800)	6% HES	Ringer's acetate	Generalized linear model	Composite of mortality or dialysis at 90 days	Higher in the 6% HES group
Enteral nutrition with eicosapentaenoic acid, g-linolenic acid and antioxidants in the early treatment of sepsis: results from a multicentre, prospective, randomized, double-	NCT00 981877	Adult patients admitted to ICU with early sepsis requiring enteral feeding (n=115)	Enteral nutrition enriched with EPA, GLA, and elevated levels of antioxidant vitamins	Isonitrogenous and isocaloric control diet (Ensure Plus)	Two-sample t-test	Development of severe sepsis or septic shock at 28 days	Lower in the enriched enteral group

blinded, controlled study: the INTERSEPT Study							
APCAP - activated protein C in acute pancreatitis: a double-blind randomized human pilot trial	NCT01 017107	Adult patients admitted to ICU with severe acute pancreatitis with one or more organ dysfunction (n=32)	Activated protein C	Placebo	Not specified	Change in SOFA score until ICU discharg e	No differe nce
A Randomized Trial of an Intensive Physical Therapy Program for Patients with Acute Respiratory Failure	NCT01 058421	Mechanically ventilated for >96 hours (n=120)	Intensive physiotherapy programme	Standard physiotherapy programme	Not specified	Continuo us Scale Physical Function al Performa nce Test (CS-PFP-10) at 30 days	No differe nce
Effect of Ganciclovir on IL-6 Levels Among Cytomegalovirus-Seropositive Adults with Critical Illness	NCT01 335932	Non-immunocompr omised adults hospitalized with respiratory failure and severe sepsis (n=160)	IV ganciclovir or oral valganciclovir	Placebo (0.9% saline)	Not specified	Serum IL-6 Level at 14 days	No differe nce
Adjunctive Glucocorticoid Therapy in Patients with Septic Shock	NCT01 448109	Adult patients with septic shock admitted to an ICU (n=3800)	Hydrocortison e 200mg/24h continuous infusion	Placebo continuous infusion	Linear regressio n model	Mortality at 90 days	No differe nce
Lower versus Higher Hemoglobin Threshold for Transfusion in Septic Shock	NCT01 485315	Adult patients admitted to ICU with septic shock and Hb 9 or less (n=998)	Liberal threshold at <9g/dL	Conservative threshold at <7g/dL	Wilcoxon signed-rank testing	Mortality at 90 days	No differe nce
Transfusion of Plasma, Platelets, and Red Blood Cells in a 1:1:1 vs a 1:1:2 Ratio and Mortality in Patients with Severe Trauma	NCT01 545232	Patients with severe trauma and major bleeding (n=680)	Transfusion 1:1:1 ratio	Transfusion 1:1:2 ratio	van Elteren test	Mortality at 30 days	No differe nce
Effect of Vitamin C Infusion on Organ Failure and Biomarkers of Inflammation and Vascular Injury in Patients with Sepsis and Severe Acute Respiratory Failure	NCT02 106975	Adult patients with sepsis and ARDS (n=170)	IV vitamin C infusion 50mg/kg every 6 hours	Placebo - 5% dextrose	Linear regressio n model	Change in modified SOFA score at 4 days	No differe nce
Immunoglobulin G for patients with necrotising soft tissue infection (INSTINCT): a randomised, blinded, placebo-controlled trial	NCT02 111161	Adult patients admitted to ICU with necrotising soft tissue infection (n=100)	IVIG 25g/day	Placebo (0.9% NaCl)	Wilcoxon signed-rank test	SF-36 function al question naire at 180 days	No differe nce
Energy-Dense versus Routine Enteral Nutrition in the Critically Ill	NCT02 306746	Mechanically ventilated adult patients (n=3997)	Energy dense (1.5kcal/ml) enteral nutrition	Routine (1kcal/ml) enteral nutrition	Mann-Whitney rank-sum test	Mortality at 90 days	No differe nce
Effect of Selective Decontamination of the Digestive Tract on	NCT02 389036	Mechanically ventilated	SDD (6h oral paste, gastric suspension	Usual care	Hierarchi cal linear	Mortality at hospital	No differe nce

Hospital Mortality in Critically Ill Patients Receiving Mechanical Ventilation		adults (n=5982)	with colistin, tobramycin, nystatin, IV antibiotics)		regression model	discharge or 90 days	
Forced fluid removal in intensive care patients with acute kidney injury: The randomised FFAKI feasibility trial	NCT02458157	Adult patients admitted to ICU with acute kidney injury and fluid accumulation of more than 10% ideal bodyweight (n=23)	Forced fluid removal (furosemide bolus then infusion or CRTT if indicated)	Usual care (CRRT discouraged unless absolute indications met)	Linear regression model	Cumulative fluid balance at 5 days	Lower in the forced fluid removal group
Early Neuromuscular Blockade in the Acute Respiratory Distress Syndrome	NCT02509078	Mechanically ventilated patients with moderate to severe ARDS (n=1008)	Cisatracurium continuous infusion and deep sedation	Usual care and lighter sedation targets	No formal statistical analysis performed	Mortality at hospital discharge or 90 days	No difference
Timing of Initiation of Renal-Replacement Therapy in Acute Kidney Injury	NCT02568722	Critically ill patients with acute kidney injury (n=3019)	Accelerated-strategy initiation of RRT	Standard-strategy initiation of RRT	No formal statistical analysis performed	Mortality at 90 days	No difference
Therapeutic Hyperthermia Is Associated<br>With Improved Survival in Afebrile Critically Ill Patients with Sepsis: A Pilot Randomized Trial	NCT02706275	Mechanically ventilated adult patients with severe sepsis (n=56)	External warming with a forced-air warming system to 1.5c greater than the lowest recorded temperature or at least 37.5c	Usual care	Non-parametric tests	HLA-DR expression at 48 hours	No difference
Early Active Mobilization during Mechanical Ventilation in the ICU	NCT03133377	Adults in ICU undergoing mechanical ventilation expected to continue beyond the calendar day after randomisation (n=750)	Early mobilisation (sedation minimisation and daily physiotherapy)	Usual care (mobilisation level usually provided in each ICU)	Median regression model	HFD at 180 days	No difference
Lower or Higher Oxygenation Targets for Acute Hypoxemic Respiratory Failure	NCT03174002	Adult patients admitted to ICU with hypoxaemic respiratory failure (n=2928)	Target PaO2 60mmHg	Target PaO2 90mmHg	van Elteren test	Mortality at 90 days	No difference
Haloperidol for the Treatment of Delirium in ICU Patients	NCT03392376	Adult patients in ICU with delirium (n=1000)	IV haloperidol 2.5mg TDS and PRN	Placebo (isotonic saline)	Linear regression model	HFD at 90 days	No difference
Early Restrictive or Liberal Fluid Management for Sepsis-Induced Hypotension	NCT03434028	Adult ICU patients with sepsis-induced hypotension refractory to initial treatment with 1 to 3 litres of intravenous fluid (n=1563)	Restrictive fluid strategy with early vasopressor use	Liberal fluid strategy	No formal statistical analysis performed	Mortality at hospital discharge or 90 days	No difference

Oxygen-Saturation Targets for Critically Ill Adults Receiving Mechanical Ventilation	NCT03 537937	Critically ill adults receiving mechanical ventilation (n=2987)	Lower oxygen target Intermediate oxygen target	Higher oxygen target	Proportio nal odds model	Ventilato r-free days at 28 days	No differe nce
Restriction of Intravenous Fluid in ICU Patients with Septic Shock	NCT03 668236	Adult patients admitted to ICU with septic shock (n=1554)	Restrictive IV fluid therapy (only if lactate >=4, MAP <50, mottling beyond edge of kneecap, oliguria, replace drain losses, maintenance fluids, input <1L)	Standard IV fluid therapy (no upper limit)	van Elteren test	Mortality at 90 days	No differe nce
Goal-directed fluid removal with furosemide versus placebo in intensive care patients with fluid overload: A randomised, blinded trial (GODIF trial, First version)	NCT04 180397	Clinically stable, adult ICU patients with at least 5% fluid overload. (n=41)	Intravenous furosemide bolus then continuous infusion	Placebo 0.9% sodium chloride	Linear regressio n model	HFD at 90 days	No differe nce
Randomized Phase 3 Trial of Ruxolitinib for COVID-19-Associated Acute Respiratory Distress Syndrome	NCT04 377620	Patients with COVID-19 associated acute respiratory distress syndrome requiring mechanical ventilation. (n=211)	Ruxolitinib 5mg or 15mg BD	Placebo	Kruskal-Wallis test	Mortality at 28 days	No differe nce
Evaluation of the Efficacy of Nicotine Patches in SARS-CoV2 (COVID-19) Infection in Intensive Care Unit Patients	NCT04 598594	Mechanically ventilated non-smoking/ vaping adults with Covid-19 pneumonia (n=220)	Transdermal nicotine patch 14mg	Placebo	Not specified	Mortality at 28 days	No differe nce
Interferon gamma-1b for the prevention of hospital-acquired pneumonia in critically ill patients: a phase 2, placebo-controlled randomized clinical trial	NCT04 793568	Critically ill patients at risk of HAP (n=109)	Recombinant interferon gamma-1b	Placebo (0.9% NaCl)	Wilcoxon rank-sum test	Composi te of HAP or mortality at 28 days	No differe nce
Effects of gabapentin on slow-wave sleep period in critically ill adult patients: a randomized controlled trial	NCT04 818450	Adult ICU patients (n=60)	Gabapentin 100mg nocte up titrated to maximum 300mg nocte	Usual care	Mann-whitney U test	Slow-wave sleep at 3 days	Increa se in gabap entin group
Intravenous aviptadil and remdesivir for treatment of COVID-19-associated hypoxaemic respiratory failure in the USA (TESICO): a randomised, placebo-controlled trial	NCT04 843761	Patients with Covid-19 associated acute hypoxaemic respiratory failure (n=471)	Aviptadil IV infusion or remdesivir IV infusion	0.9% sodium chloride IV infusion	Proportio nal odds model	Recovery categoriz ed by a six-category ordinal outcome at 90 days	No differe nce
Early and sustained Lactobacillus plantarum probiotic therapy in critical illness: the	ACTRN 126170 007833 25	Adult ICU patients After one third of enrolment	L. planatarum 299v one capsule OD	Placebo	Wilcoxon rank-sum test	HFD at 60 days	No differe nce

randomised, placebo-controlled, restoration of gut microflora in critical illness trial (ROCIT)		complete, ethical permission to recruit patients without capacity rescinded, leading to low severity of illness (5% mortality) (n=221)					
Iron and erythropoietin to heal and recover after intensive care (ITHRIVE): A pilot randomised clinical trial	ACTRN 126210 005958 19	Adults with anaemia (haemoglobin <100 g/L), requiring ICU-level care for more than 48h, and likely to be ready for ICU discharge within 24h. (n=40)	Single dose of 1g intravenous ferric carboxymaltose infusion over two hours in 100ml of 0.9% saline, and a single dose of intravenous Epoetin alfa 40,000 IU	Placebo (0.9% NaCl)	Quantile regression model	Feasibility measures at 28 days	Demonstrated feasibility
The Effect of a Liberal Approach to Glucose Control in Critically Ill Patients with Type 2 Diabetes	ACTRN 126160 011354 04	Critically ill patients with pre-existing type 2 diabetes (n=434)	Liberal glucose control 180-252 mg/dl	Usual care with glucose target 108-180 mg/dl	Not specified	Incidence of hypoglycaemia at 28 days	Reduced incidence in liberal glucose target group
A Phase II Cluster-Crossover Randomized Trial of Fentanyl versus Morphine for Analgosedation in Mechanically Ventilated Patients	ACTRN 126190 009391 90	Mechanically ventilated adult ICU patients (n=737)	Fentanyl continuous infusion for analgo-sedation	Morphine continuous infusion for analgo-sedation	Quantile regression model	Ventilator-free days at 28 days	Increase in fentanyl group
Acetaminophen for Fever in Critically Ill Patients with Suspected Infection	ACTRN 126110 009819 21 ACTRN 126120 005138 19	Adult patients in ICU with fever and known or suspected infection (n=700)	IV paracetamol 1g/ 100ml every 6 hours	IV 5% dextrose 100ml every 6 hours	Wilcoxon rank-sum test	ICU-Free Days at 28 days	No difference
Remi-fent 1 - A pragmatic randomised controlled study to evaluate the feasibility of using remifentanil or fentanyl as sedation adjuncts in mechanically ventilated patients	ACTRN 126200 007199 32	Mechanically ventilated adult patients admitted to the study ICU (n=212)	Remifentanil continuous infusion	Fentanyl continuous infusion	Mann-Whitney test	Feasibility measures at 28 days	Demonstrated feasibility

Supplementary Table S5. Table of frequencies of variations of HFD outcome name in included studies

Outcome Name	Number (%)
Hospital-free days	63 (57.3)
Days alive out of the hospital	26 (23.6)
Days alive outside of hospital	5 (4.5)
Days alive and free of hospitalization	4 (3.6)
Days alive at home	3 (2.7)
Days outside of hospital	2 (1.8)
Days alive after hospital discharge	2 (1.8)
Days free of the index hospital and alive	1 (0.9)
Days alive and not in hospital	1 (0.9)
Days not in hospital	1 (0.9)
Hospital length of stay (variation of)	1 (0.9)
Institution-free days	1 (0.9)

Supplementary Table S6. Table of included studies with HFD as a primary outcome showing features of the definition of HFD

Trial Name	Trial Status	MCID	Duration of HFD follow-Up (days)	Handling of mortality during follow-up.	Included As a Hospital-Day	Included as Hospital-Free Day
Early Active Mobilization during Mechanical Ventilation in the ICU (TEAM)	Published manuscript with results (completed)	7 days	180	Mortality allocated 0 HFD.[1]	Acute care hospital<br>Nursing home<br>LTACH	Anything else
Haloperidol for the Treatment of Delirium in ICU Patients (AID-ICU)	Published manuscript with results (completed)	8% (4.7 days)	90	Actual number of HFD used.[2]	Acute care hospital	Nursing home<br>LTACH
Haloperidol vs. placebo for the treatment of delirium in ICU patients: a pre-planned, secondary Bayesian analysis of the AID-ICU trial	Published secondary analysis with results (completed)	1 day	90	Actual number of HFD used.[2]	Acute care hospital	Nursing home<br>LTACH<br>(As per AID-ICU)
Goal-directed fluid removal with furosemide versus placebo in intensive care patients with fluid overload: A randomised, blinded trial (GODIF trial, First version)	Published manuscript with results (completed)	8%	90	Actual number of HFD used.[2]	Acute care hospital	Nursing home<br>LTACH
Furosemide versus placebo for fluid overload in intensive care patients, The randomised GODIF trial second version: Statistical analysis plan	Published protocol (recruiting)	8% (4.7 days)	90	Actual number of HFD used.[2]	Acute care hospital	Nursing home<br>LTACH
Study protocol for TARGET protein: The effect of augmented administration of enteral protein to critically ill adults on clinical outcomes: A cluster randomised, cross-sectional, double cross-over, clinical trial	Published protocol (completed recruitment)	1 day	90	Death allocated 0 HFD.[1]	Only the 'index hospital' is considered as hospital	Anything else
Iron and erythropoietin to heal and recover after intensive care (ITHRIVE): A pilot randomised clinical trial	Published manuscript with results (completed)	3 days	90	Actual number of HFD used.[1]	Acute care hospital<br>Nursing home<br>LTACH	Anything else

Plasma Exchange (PLEX) and Convalescent Plasma (CCP) in COVID-19 Patients with Multiorgan Failure (NCT04634422)	Trial registration (unknown status)	7.31 days	90	Not specified	Not specified	Not specified
Early and sustained Lactobacillus plantarum probiotic therapy in critical illness: the randomised, placebo-controlled, restoration of gut microflora in critical illness trial (ROCIT)	Published secondary analysis with results (completed)	4 days	60	Death allocated 0 HFD.[1]	Acute care hospital<br>Nursing home<br>LTACH	Anything else
Fibrinogen Early In Severe Trauma StudY II (FEISTY II) (NCT05449834)	Trial registry	Not specified	90	Not specified	Not specified	Not specified
Balanced Multi-Electrolyte Solution Versus Saline Trial for Diabetic KetoAcidosis (BEST-DKA) (NCT05752279)	Trial registry	Not specified	28	Not specified	Not specified	Not specified
Randomised, controlled, feasibility trial comparing vasopressor infusion administered via peripheral cannula versus central venous catheter for critically ill adults: a study protocol (VIPCA)	Published protocol or statistical plan	Not specified	30	Not specified	Not specified	Not specified

MCID, Minimum clinically important difference; RCT, randomized controlled trial; LTACH, long term acute care hospital.

[1] If patient dies after discharge from the index hospitalisation but during the follow-up period, no HFD prior to death were counted so they are assigned a value of 0 HFD. If a patient dies during the index hospitalisation, they are also assigned 0 HFD.

[2] If a patient after discharge from the index hospitalisation but during the follow-up period, the actual number of HFD accumulated before their death is used. If a patient dies during the index hospitalisation, they are assigned 0 HFD.

Supplementary Table S7. Recommendations for the use of HFD in critical care research (with justification)

Area	Recommendation	Justification
Definition and Reporting	The HFD outcome should be clearly and prospectively defined in the trial registration or protocol of a study.	The numerous nuances to HFD can make substantial difference in its interpretation and external validity.
	Mortality and hospital length of stay in the same follow-up period should be reported.	HFD is a composite outcome. An intervention may have bi-directional effects e.g decrease mortality but increase length of stay.
Mortality	How mortality is used in the calculation of HFD should be prospectively specified.	This can make substantial changes to the overall HFD value and is important to the external validity and for any meta-analysis.
	More PPI work is needed to consider different values assigned to mortality. In the meantime, we suggest assigned a value of 0 HFD is assigned.	This is in line with most studies which have used HFD as an outcome, and the ANZICS Clinical Trials Group recommendation.[22]
Definition of a Hospital-Free Day	What was counted as a 'hospital-day' should be clearly specified.	This will affect the patient-centredness and external validity of the outcome.
	We suggest that the calculation of HFD should include time spent in an LTACH and ED visits, as well as nursing home.	This is in line with recent engagement with stakeholders.[24] Other PPI work has demonstrated the value that patients place on being discharged home.[12]
	Authors should consider using institution-free days which typically include outpatient visits and other healthcare encounters in the calculation.	More PPI work is needed to consider whether institution-free days are a more patient-centred outcome overall than HFD.
Quality-Weighted HFD	More work is needed to validate quality-weighted HFD.	Early work has suggested that it may be more preferred by stakeholders to HFD.[25] However, it has yet to be validated in this population.
Readmissions	We strongly recommend that follow-up should not be censored on discharge from the index hospitalisation. An outcome which is censored on discharge from the index hospital admission should not be identified as HFD.	An HFD outcome that is censored on discharge from the index hospitalisation would only consider index hospitalisation length of stay and index in-hospital mortality during the follow-up period. Mortality and readmissions in the period following the index hospitalisation are of significant importance to patients and should be reflected in the outcome.
	We recommend that readmissions to an acute-care hospital should be included in the calculation of HFD.	Readmissions add to the total hospital length of stay in the follow-up period and are important in evaluating an intervention.

		However, we recognise the nuances surrounding this and the desire to only capture readmissions relevant to the index hospitalisation or intervention.
Follow-Up Duration	Follow-up duration should be sufficient to allow discharge from the index hospitalisation for the majority of patients and to capture relevant readmissions.	The ideal follow-up duration will vary between different patient populations and interventions but requires careful consideration. The majority of included studies have used 90 days.
MCID	The MCID should be prospectively specified and justified. More work is needed to consider the MCID at different follow-up durations.	We commend the PPI work that has taken place to gain consensus on an appropriate MCID for HFD. We suggest that this work is considered when deciding an MCID in future trials.
Statistical Analysis	We suggest that the analysis method used reflects the distribution of the HFD data and is described and justified in sufficient detail. More work in this area is required to establish the optimal methods of analysis.	The statistical analysis of HFD is complex and will vary depending on the definition used. Further work should compare simpler parametric and non-parametric methods with using more complex statistical models.

HFD, Hospital free days; PPI, patient and public involvement; LTACH, long-term acute care hospital; ED, emergency department; MCID, minimal clinically important difference
